# Genetic grouping and geographic distribution of Piscine orthoreovirus-1 (PRV-1) in farmed Atlantic salmon in Norway

**DOI:** 10.1186/s13567-021-01000-1

**Published:** 2021-10-14

**Authors:** Nina A. Vatne, Marit Stormoen, Morten Lund, Magnus Devold, Espen Rimstad, Øystein Wessel

**Affiliations:** 1 Faculty of Veterinary Medicine, Norwegian University of Life Sciences, 1433 Ås, Norway; 2 PatoGen AS, 6002 Ålesund, Norway

**Keywords:** PRV-1, heart and skeletal muscle inflammation, virulence, Atlantic salmon, genogroups

## Abstract

Piscine orthoreovirus-1 (PRV-1) is the causative agent of heart and skeletal muscle inflammation (HSMI) in farmed Atlantic salmon (*Salmo salar*). However, it has been shown that PRV-1 variants differ in their ability to induce HSMI. The objective of this work was to identify the PRV-1 variants in Norwegian aquaculture and their geographical distribution. Sequencing and subsequent analysis of the five genomic segments (S1, S4, M2, L1 and L2) putatively linked to virulence, made out the basis of the study. Thirty-seven Norwegian PRV-1 isolates were sequenced, and they grouped into eight genogroups based on combinations of the five analyzed genomic segments. Two groups were defined as high-virulent and two low-virulent, based on comparison with PRV-1 reference isolates with known virulence. The remaining four groups were of unknown virulence. The geographic distribution indicated a higher frequency of the high-virulent isolates in the mid- and northern regions. The present study confirms circulation of both high- and low-virulent isolates of PRV-1 in farmed Atlantic salmon in Norway. To reduce the impact of PRV-1 related disease, detection and differentiation between high- and low-virulent genogroups of PRV-1 could be a targeted approach for reduction of high-virulent variants.

**Supplementary Information:**

The online version contains supplementary material available at 10.1186/s13567-021-01000-1.

## Introduction

Piscine orthoreovirus is a widespread virus in salmonid fish, with three strains PRV-1, PRV-2, PRV-3, which are associated with different diseases in different salmonid species [1]. The PRV-1 is the causative agent of heart and skeletal muscle inflammation (HSMI) in farmed Atlantic salmon (*Salmo salar*). The virus can be found in fish from farms with HSMI outbreak as well as in farms where the disease has not been observed [2, 3]. It has been shown that PRV-1 variants differ in their ability to induce HSMI [4], and there is a need for further characterization of the different variants and map their distribution.

HSMI typically occurs during the first year in sea water, often 5–7 months after the fish have been transferred to the sea [5]. Pale and anorexic fish with abnormal swimming behavior are common clinical observations. The pathological lesions include inflammation and necrosis of myocardium and red skeletal muscle, and these lesions are used to set a diagnosis [6]. HSMI was first described in 1999 [7], and today more than 100 outbreak are reported in Norway annually [8]. However, this could be an underestimation as HSMI was removed from the list of notifiable diseases in Norway 2014 and thus no longer mandatory to report. Outbreaks seem to be most prevalent in mid- and northern-Norway, and less prevalent in the western and southern regions despite a more intensive farming [8]. HSMI has also been reported in Scotland, Chile, and Canada [9–11]. However, in Canada as well as Faroe Islands, little clinical disease has been identified, although the virus is prevalent [12, 13]. PRV-1 is relatively widespread in wild Atlantic salmon and there appears to be transmission of PRV between farmed and wild salmon [14, 15]. However, HSMI has not been reported from wild fish.

PRV is a naked virus with an icosahedral particle encompassing a ten-segmented, double-stranded RNA (dsRNA) genome [2]. The genome segments sort into three groups: small (S1–S4), medium (M1–M3) and large (L1–L3) which encodes for at least 11 proteins; 8 structural, and 3 non-structural [7]. Segment S1 has been extensively used for phylogenetic analysis of PRV-1 [14, 16–18]. Analysis of segment S1 groups PRV-1 into two main monophyletic clades, which has been referred to as sub-genotype Ia and Ib [16, 17]. Other studies have reported four S1 groups (I–IV), where group I is consistent with Ib and group II, II and IV are comprised within Ia [14, 18]. The genetic variability seems to be rather high in Norway including variants of both Ia and Ib, whereas the isolates of Western North America are genetically homogenous clustering only to group Ia [19]. The studies focusing on the nucleotide sequence of S1 have contributed in the mapping of the geographical distribution of the genetic groups. However, they have not been able to define virulence properties of the virus.

A segmented RNA virus like PRV employs many strategies, including mutations, recombination and reassortment of genomic segments to increase their fitness and evolve their genomes in response to selection pressures of changing environments. RNA viruses in general have high mutation rates, but single-stranded viruses mutate faster than double-strand viruses, and genome size correlates negatively with mutation rate [20]. For segmented viruses such as the reoviruses reassortment is an important driver of genetic variation [21]. Previous studies have suggested reassortment as a common mechanism for evolution of isolates of *Mammalian orthoreoviruses* with different virulence properties [22–24].

The presence of virulence differences between PRV-1 variants have been hypothesized based on field observations and challenge trials. Notably, a high number of HSMI outbreaks in farmed Atlantic salmon has been reported in Norway in contrast to a limited number in Canada despite widespread detection of the virus in both countries [8, 11]. Furthermore, challenge trials with Norwegian PRV-1 isolates have repeatedly reproduced HSMI lesions [3, 25, 26], whereas trials using Canadian isolates have produced little or no cardiac lesions [12, 13]. Although these contradictory results are highly indicative of virulence differences, they cannot be used as conclusive evidence. Virulence is defined as the relative capacity to cause damage, in this case HSMI, and is always measured relative to a standard, often to another variant of the virus. The putative high and low virulent PRV-1 variants had been tested in separate but not in joint trials. The lack of standardization with respect to dose, host and environmental factors prevented a conclusion that the variation in disease outcome was determined by virulence differences. However, the hypothesized virulence difference was finally confirmed in a dose standardized challenge trial demonstrating that PRV-1 isolates differ in their ability to induce the heart lesions typical of HSMI [4]. That study compared several PRV-1 isolates in a common trial including two Norwegian field isolates (NOR-2018 SF, NOR-2018 NL), three historical Norwegian isolates (NOR-1988, NOR-1996, NOR-1997) and one isolate from British Colombia, Canada (CAN 16-005ND). All isolates included in that study induced heart lesions, but the Norwegian 2018 isolates were shown to be of higher virulence inducing severe cardiac lesions consistent with HSMI [4]. The confirmation of high and low virulent isolates prompts further phenotypic characterization of the viruses.

Virulence differences between virus isolates are reflected in the viral genome. Previous studies have identified segments S1 and M2 as important determinants for PRV-1 virulence, with a likely co-evolution for these two segments [19]. S1 encodes for σ3, while M2 encodes for the µ1 protein [7]. Together these two proteins form a heterohexamer in the outer capsid of the virus particle [7]. Sequence analysis comparing PRV-1 variants of high and low virulence revealed that while the σ3 and µ1 proteins likely play important roles in development of disease, other segments must also contribute to virulence. This is illustrated through the isolate known as NOR-1997, which despite having S1 and M2 segments identical to the high-virulent isolates do not induce pathological changes consistent with HSMI. Based on phylogenetic analysis, and the grouping of high- and low-virulent isolates, the segments S4, L1 and L2 were suggested as other potential determinants for virulence [4]. The function of the proteins encoded by the S4, L1 and L2 segments have been inferred from studies focusing on putatively encoded proteins and their similarity with proteins encoded from mammalian reoviruses. L1 codes for the λ3 protein which is the RNA-dependent RNA polymerase responsible for viral translation and replication, while L2 codes for λ2, which is both a structural protein as well as a capping enzyme [7]. The S4 segment codes for σ1, the cell attachment protein [7].

The objective of this work was to identify the different PRV-1 variants in Norwegian aquaculture and map their geographical distribution based on sequencing and analysis of the five genomic segments (S1, S4, M2, L1 and L2) putatively linked to virulence.

## Materials and methods

### Samples

Samples collected from Norwegian farmed Atlantic salmon were used to obtain 37 partial genome sequences of PRV-1 (Table 1). The material was collected from samples sent to PatoGen AS (Ålesund, Norway) for PRV-1 screening. PatoGen AS is a real-time PCR analysis company accredited according to international standard ISO 17025. Each producer was contacted to get approval for performing the sequencing of the PRV-1 isolates and access to meta data for the isolate. Thirty-five samples were collected in 2019, one sample was collected in 2017 and one in 2016. The samples originated from a wide geographical range and 12 of the 13 Norwegian aquaculture production zones were represented in the collection. Twenty-eight samples were collected from sea pens and nine from freshwater facilities. Thirty-one of the samples were from the heart and six from the kidney (Table 1). The method used for RNA extraction and Real Time PCR testing for the presence of PRV-1 was PatoGen Analyse’s in‐house methods described previously [27].

**Table.1:** **PRV-1 isolates sequenced in the study**

Name	Sample date	Prod. zone	Organ	Ct-value	Sequencing method	Accession number
NOR2019 V-AGD-1737m	02.09.19	1	Heart	28.1	Amplicon	MW831860–MW831864
NOR2019 ROG-1666m	09.07.19	2	Heart	22.0	Amplicon	MW831785–MW831789
NOR2016 ROG-1661s	27.09.16	2	Heart	23.9	Amplicon	MW831680–MW831684
NOR2019 ROG-1751m	28.01.19	2	Heart	28.9	Amplicon	MW831790–MW831794
NOR2019 HRD-1523m	19.02.19	3	Heart	16.5	Amplicon	MW831690–MW831694
NOR2019 HRD-1525m	12.11.19	3	Heart	20.1	Amplicon	MW831715–MW831719
NOR2019 HRD-1744m	08.01.19	3	Heart	28.8	Amplicon	MW831725–MW831729
NOR2019 SFJ-1674m	06.08.19	4	Heart	27.4	Amplicon	MW831795–MW831799
NOR2017 SFJ-1660s	27.03.17	4	Kidney	23.4	Amplicon	MW831685–MW831689
NOR2019 HRD-1743m	23.09.19	4	Heart	21.8	Amplicon	MW831720–MW831724
NOR2019 SFJ-1745m	04.07.19	4	Heart	28.8	Amplicon	MW831805–MW831809
NOR2019 SFJ-1736m	07.03.19	4	Heart	28.1	Amplicon	MW831800–MW831804
NOR2019 MRO-5075s	10.09.19	5	Kidney	20.6	Amplicon	MW831740–MW831744
NOR2019 MRO-5072s	21.03.19	5	Heart	18.6	WGS	MW831735–MW831739
NOR2019 MRO-5088m	08.08.19	5	Heart	20.4	WGS	MW831750–MW831754
NOR2019 TRL-1675m	08.01.19	6	Heart	26.5	Amplicon	MW831815–MW831819
NOR2019 MRO-1665m	30.01.19	6	Heart	22.2	Amplicon	MW831730–MW831734
NOR2019 MRO-5087m	30.01.19	6	Heart	18.6	WGS	MW831745–MW831749
NOR2019 TRL-1765m	13.06.19	6	Heart	30.5	Amplicon	MW831820–MW831824
NOR2019 TRL-1664m	18.07.19	7	Heart	23.8	Amplicon	MW831810–MW831814
NOR2019 TRL-5070s	12.07.19	7	Heart	16.4	WGS	MW831825–MW831829
NOR2019 TRL-5091m	09.04.19	7	Heart	17.4	WGS	MW831830–MW831834
NOR2019 NRL-5078m	20.03.19	8	Heart	20.5	Amplicon	MW831780–MW831784
NOR2019 NRL-1750m	11.07.19	8	Heart	29.1	Amplicon	MW831765–MW831769
NOR2019 NRL-1676m	16.05.19	9	Heart	21.7	Amplicon	MW831755–MW831759
NOR2019 NRL-1742m	21.08.19	9	Heart	21.4	Amplicon	MW831760–MW831764
NOR2019 NRL-5071s	12.08.19	9	Kidney	16.7	WGS (Amplicon L1)	MW831770–MW831774
NOR2019 NRL-5073s	09.04.19	9	Kidney	18.8	WGS	MW831775–MW831779
NOR2019 TRO-1677m	07.05.19	10	Heart	26.3	Amplicon	MW831840–MW831844
NOR2019 TRO-5077m	09.10.19	10	Heart	19.6	WGS (Amplicon L1 + L2)	MW831850–MW831854
NOR2019 TRO-1662s	14.10.19	11	Kidney	24.9	Amplicon	MW831835–MW831839
NOR2019 TRO-5089m	04.07.19	11	Heart	15.9	WGS	MW831855–MW831859
NOR2019 TRO-1735m	07.02.19	11	Heart	27.6	Amplicon	MW831845–MW831849
NOR2019 FNM-5090m	10.08.19	12	Heart	15.4	WGS (Amplicon L1)	MW831710–MW831714
NOR2019 FNM-1527m	10.04.19	12	Heart	20.9	Amplicon	MW831695–MW831699
NOR2019 FNM-1642s	25.04.19	12	Kidney	25.4	Amplicon	MW831700–MW831704
NOR2019 FNM-1817m	18.02.19	12	Heart	17.2	Amplicon	MW831705–MW831709

### Sequencing

Next-Generation Sequencing was performed using the Ion Total RNA-Seq Kit v2 library preparation kits (Thermo Fisher Scientific, Waltham, MA, USA) following the manufacturers recommendations. The library preparation was setup up on an Ion Chef system and the samples were sequenced on Ion S5™System from (Thermo Fisher Scientific). Most of the samples (27/37) were sequenced using amplicon sequencing. The remaining samples (10/37) were sequenced using whole genome sequencing (WGS), but for three of these samples, L1, L2 or both segments were re-sequenced using amplicon sequencing due to poor coverage on WGS.

### Reference isolates

Six reference isolates with previously determined virulence were used in the analysis [4]. Two of the reference isolates had been characterized as high-virulent and originated from farmed Atlantic salmon collected in 2018 (NOR-2018 SF and NOR-2018 NL). Four of the reference isolates had been characterized with low virulence. These included tree historical Norwegian isolates pre-dating the discovery of HSMI in Norway (NOR-1988, NOR-1996 and NOR-1997) and a Canadian isolate collected in British Colombia (CAN 16-005ND).

### Sequence and phylogenetic analysis

Multiple sequence alignments were performed using AlignX (Vector NTI Advance 11.0) and Mega X software v.10.17 (available from www.megasoftware.net). Phylogenetic analysis was performed with Mega X, using partial-length nucleotide sequences from five gene segments: S1, M2, L1, L2 and S4 (Table 2). Phylogenetic analysis was also performed on six deduced amino acid sequences encoded by these five segments (σ3, p13, µ1, λ2, λ3 and σ4). Maximum Likelihood (ML) was used to generate phylogenetic trees for nucleotide sequences, and neighbor joining (NJ) for amino acid sequences. For all trees, the best-fitting substitution models suggested by the software were used. Bootstrap values were calculated from 1000 replicates, and values above 70 considered significant.

**Table.2:** **Overview of partial sequences analyzed**

Segment (protein)		Partial coding sequence bp (aa)	
S1	(σ3)	77–1021	(70–330)
	(p13)		(1–123)
M2	(µ1)	92–2108	(47–687)
L1	(λ3)	2453–3877	(827–1286)
L2	(λ2)	2332–3902	(809–1290)
S4	(σ1)	61–1006	(69–315)

### Nomenclature of phylogenetic groups and characterization of virus isolates

In the present study, we analyzed the five segments linked to virulence (S1, S4, M2, L1 and L2) focusing on the amino acid sequence. For each segment the isolates were assigned to either Group A or B, i.e., S1 (σ3) Group A or B, L1 (λ3) Group A or B etc., based on phylogenetic analysis of the amino acid sequence of the encoded proteins. Thereafter, the virus isolates were grouped into genogroups (High, Low, Unknown) based on the combinations of the five segments compared to those of the reference isolates. The grouping on segment S1 in the present study into S1 (σ3) Group A or B correspond to Ia and Ib of the established classification system [16, 17]. However, the classification of the present study takes into account the combination of all five segments linked to virulence which is the basis for the genogroups (High, Low, Unknown).

## Results

The five genomic segments (S1, S4, M2, L1 and L2) putatively linked to virulence were sequenced from thirty-seven PRV-1 isolates collected from farmed Atlantic salmon in Norway. Phylogenetic analysis was performed on each separate segment, which in turn was used to characterize genogroups of virus present in farmed Atlantic salmon in Norway.

### S1 (σ3) and M2 (µ1) clustered into two distinct phylogenetic groups

**Figure 1:**
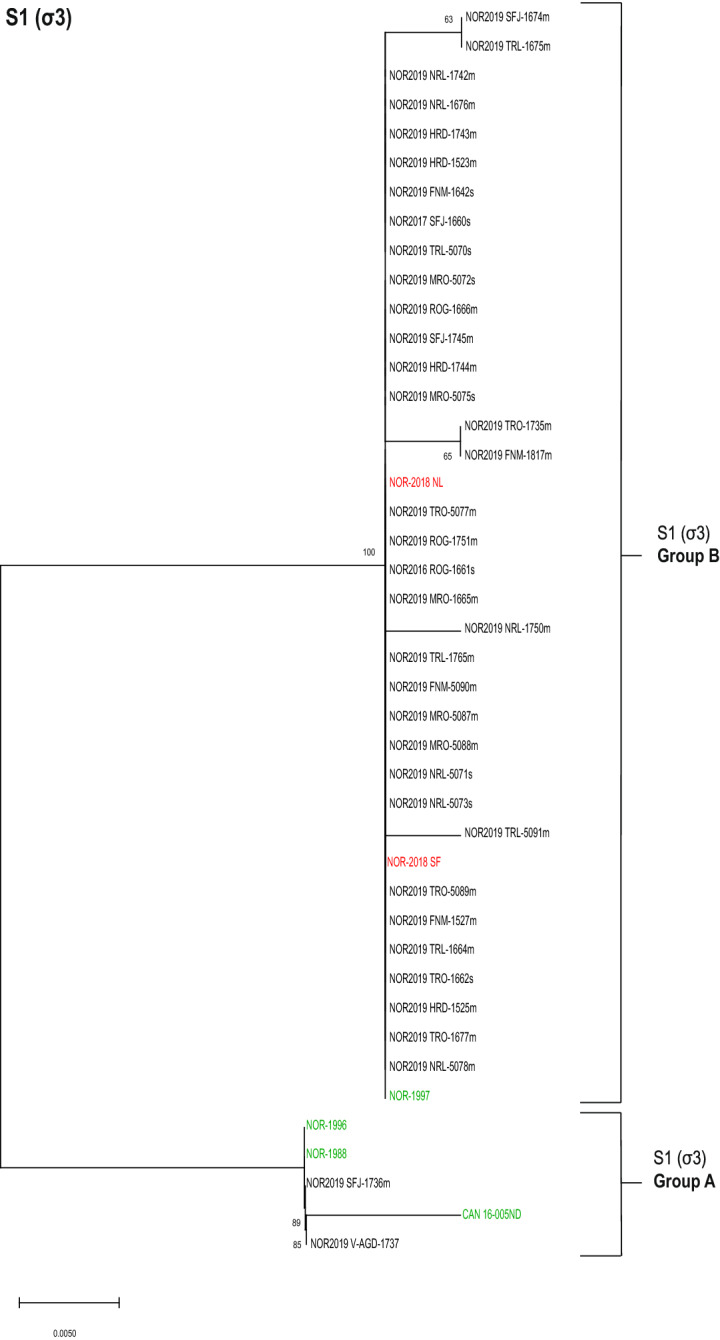
**Phylogenetic analysis of S1 (σ3).** Phylogenetic tree constructed from partial sequences of σ3 (aa 70–330) using neighbor joining (NJ). The analysis included 37 field isolates (black) and six reference isolates of known virulence (high virulent in red, low virulent in green). The isolates clustered into two distinct groups annotated as S1 (σ3) Group A and Group B. Bootstrap values were calculated from 1000 replicates and values above 70 were considered significant.

Phylogenetic analysis of the partial amino acid sequence of σ3 (segment S1) clustered the PRV-1 isolates into two distinct groups (Figure 1). Thirty-five of 37 isolates clustered to one group, hereafter referred to as S1 (σ3) Group B. These isolates had identical or highly similar sequences to reference isolates confirmed with high virulence (NOR-2018 NL and NOR-2018 SF). However, the S1 (σ3) Group B also included a reference isolate of known low-virulence, the NOR-1997 isolate. The results suggest that most PRV-1 isolates found in the Atlantic salmon production in Norway today cluster to the S1 (σ3) Group B. The remaining two isolates grouped with reference isolates confirmed with low virulence (NOR-1996, NOR-1988 and CAN-16005ND), hereafter known as S1 (σ3) Group A (Figure 1). The two groups differed by nine amino acids within the analyzed sequence (Additional file 1). Single amino acid substitutions in addition to those nine amino acids defining the grouping were observed for both reference- and field isolates, but these amino acid variations did not impact the phylogenetic clustering of the isolates. A similar tree topology was observed by phylogenetic analysis of amino acid sequence of p13, a virus protein also encoded by S1 (Additional file 2), as well as of the nucleotide sequence of segment S1 (Additional file 3).

**Figure 2:**
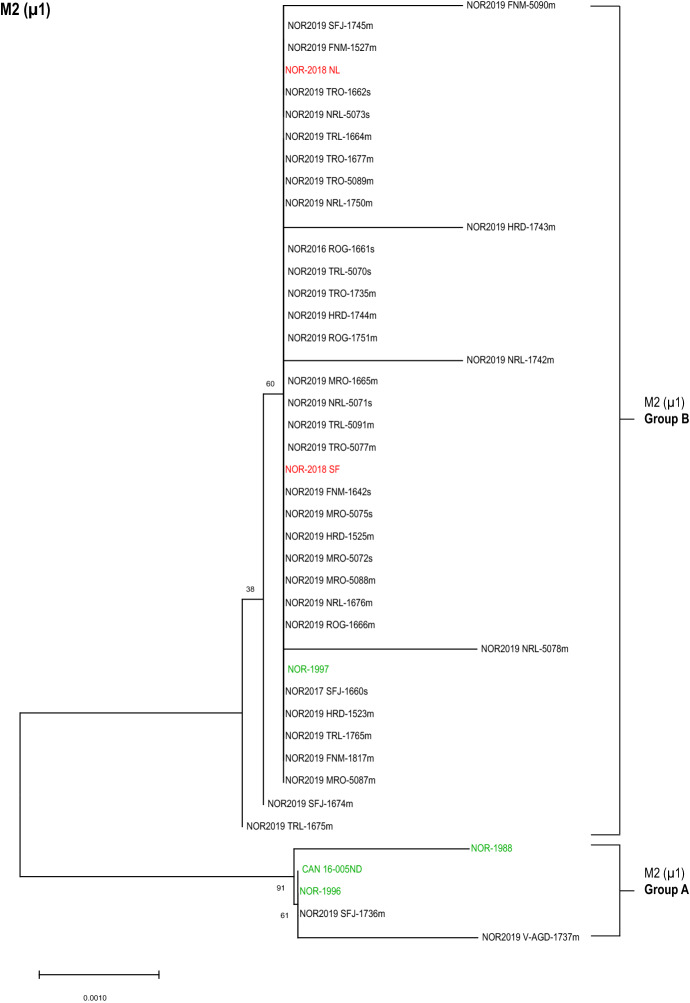
**Phylogenetic analysis of M2 (µ1).** Phylogenetic tree constructed from partial sequences of µ1 (47 – 687) using neighbor joining (NJ). The analysis included 37 field isolates (black) and six reference isolates of known virulence (high virulent in red, low virulent in green). The isolates clustered into two distinct groups annotated as M1 (µ1) Group A and Group B. Bootstrap values were calculated from 1000 replicates and values above 70 were considered significant.

The phylogenetic analysis of the partial amino acid sequence of µ1 (segment M2) adhered to the same pattern as S1 (σ3), dividing the isolates into the same two distinct groups (Figure 2). All isolates clustered to the same phylogenetic groups for σ3 and µ1, i.e., Groups A and B contained identical isolates for both proteins. The M2 (µ1) Group B, which included the high-virulent reference isolates NOR-2018 NL and NOR-2018 SF, as well as low-virulent reference isolate NOR-1997, was dominating. The same two field sequences that clustered with the low-virulent group (consisting of NOR-1996, NOR-1988 and Can 16-005ND) for σ3 also clustered with these for µ1, thus belonging to the M2 (µ1) Group A. Three amino acids differed between the two groups in the partial sequence analyzed (Additional file 1). Single amino acid substitutions besides those defining the groups were present but did not impact the phylogenetic clustering of the isolates. A similar tree topology was observed by phylogenetic analysis of the nucleotide sequence of segment M2 (Additional file 4).

### Analysis of L1 (λ3) revealed two distinct phylogenetic groups

**Figure 3:**
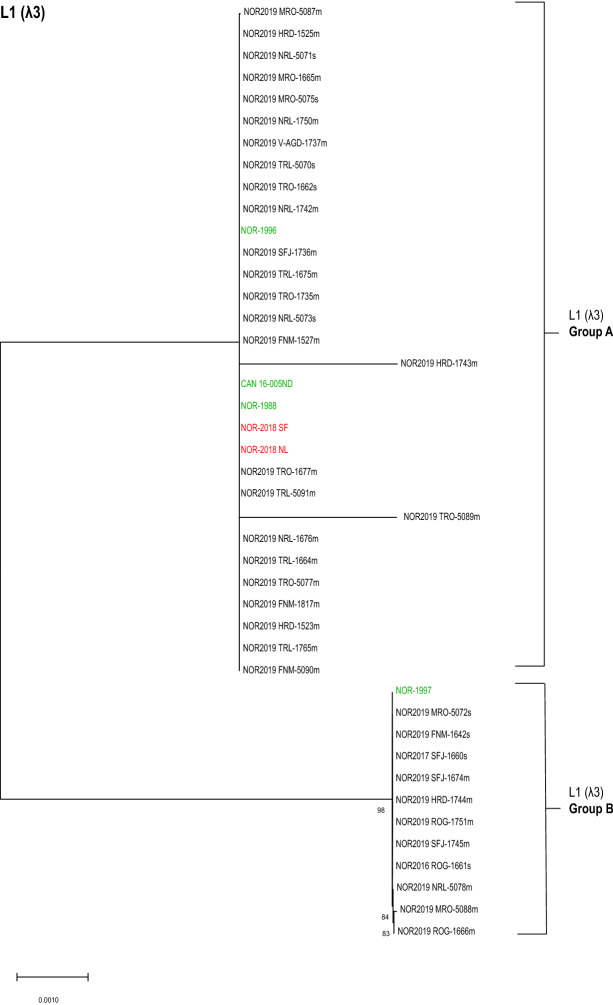
**Phylogenetic analysis of L1 (λ3).** Phylogenetic tree constructed from partial sequences of λ3 (aa 827–1286) using neighbor joining (NJ). The analysis included 37 field isolates (black) and six reference isolates of known virulence (high virulent in red, low virulent in green). The isolates clustered into two distinct groups annotated as L1 (λ3) Group A and Group B. Bootstrap values were calculated from 1000 replicates and values above 70 were considered significant.

The phylogenetic analysis of the partial amino acid sequence of λ3 (segment L1) separated the isolates in two distinct groups hereafter known as L1 (λ3) Group A and B (Figure 3). Importantly, these two distinct phylogenetic groups were composed of different isolates than those observed for S1 (σ3)- and M2 (µ1) Groups A and B. Eleven out of 37 isolates clustered with the low-virulent reference isolate NOR-1997; L1 (λ3) Group B. Twenty-six out of 37 clustered with the other five reference isolates, which included both high- and low-virulent isolates; L1 (λ3) Group A. This indicate that both variants of the λ3 protein are prevalent in Norwegian Atlantic salmon farming today. Four amino acids within the analyzed region were the difference hallmark between the two groups. Other single amino acid substitutions within the group did not impact on the phylogenetic clustering of the isolates. A similar tree topology was observed by phylogenetic analysis of the nucleotide sequence for L1 (Additional file 5).

### Analysis of L2 (λ2) revealed two distinct phylogenetic groups

**Figure 4:**
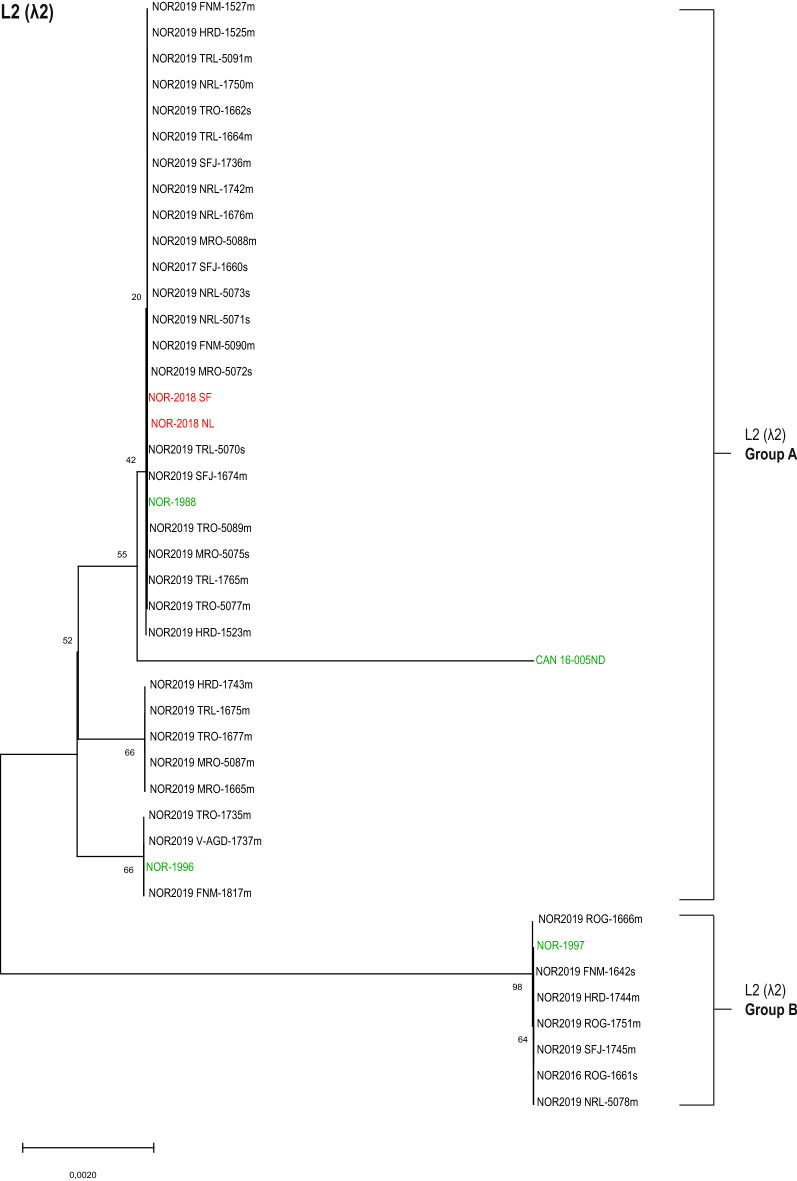
**Phylogenetic analysis of L2 (λ2).** Phylogenetic tree constructed from partial sequences of λ2 (aa 809–1290) using neighbor joining (NJ). The analysis included 37 field isolates (black) and six reference isolates of known virulence (high virulent in red, low virulent in green). The isolates clustered into two distinct groups annotated as L2 (λ2) Group A and Group B. Bootstrap values were calculated from 1000 replicates and values above 70 were considered significant.

Analysis of the partial sequence of λ2 (segment L2) also grouped the virus isolates in two main groups (Figure 4). Notably, these two distinct phylogenetic groups had a different composition of isolates than that observed for S1 (σ3), M2 (µ1) and L1 (λ3). Seven out of the 37 isolates grouped with the low-virulent NOR-1997 reference isolate; here called L2 (λ2) Group B. Thirty out of 37 isolates grouped with five reference isolates which included both high- and low-virulent reference isolates; here called L2 (λ2) Group A. Five amino acids within the region analyzed differed between the two groups. Furthermore, a tendency for two subgroupings within L2 (λ2) Group A was observed, although bootstrap value support for this clustering was weak (<  70). No covariation was seen between L1 (λ3) and L2 (λ2), in contrast to that of S1 (σ3) and M2 (µ1). More single amino acids substitutions were present in the λ2 protein than for the other virus proteins in our study (Additional file 1). These amino acid differences contributed to the subgrouping seen in the phylogenetic analysis of this segment. A similar tree topology was observed by phylogenetic analysis of the nucleotide sequence of segment L2 (Additional file 6).

### Two variable amino acid positions within σ1 in segment S4

**Figure 5:**
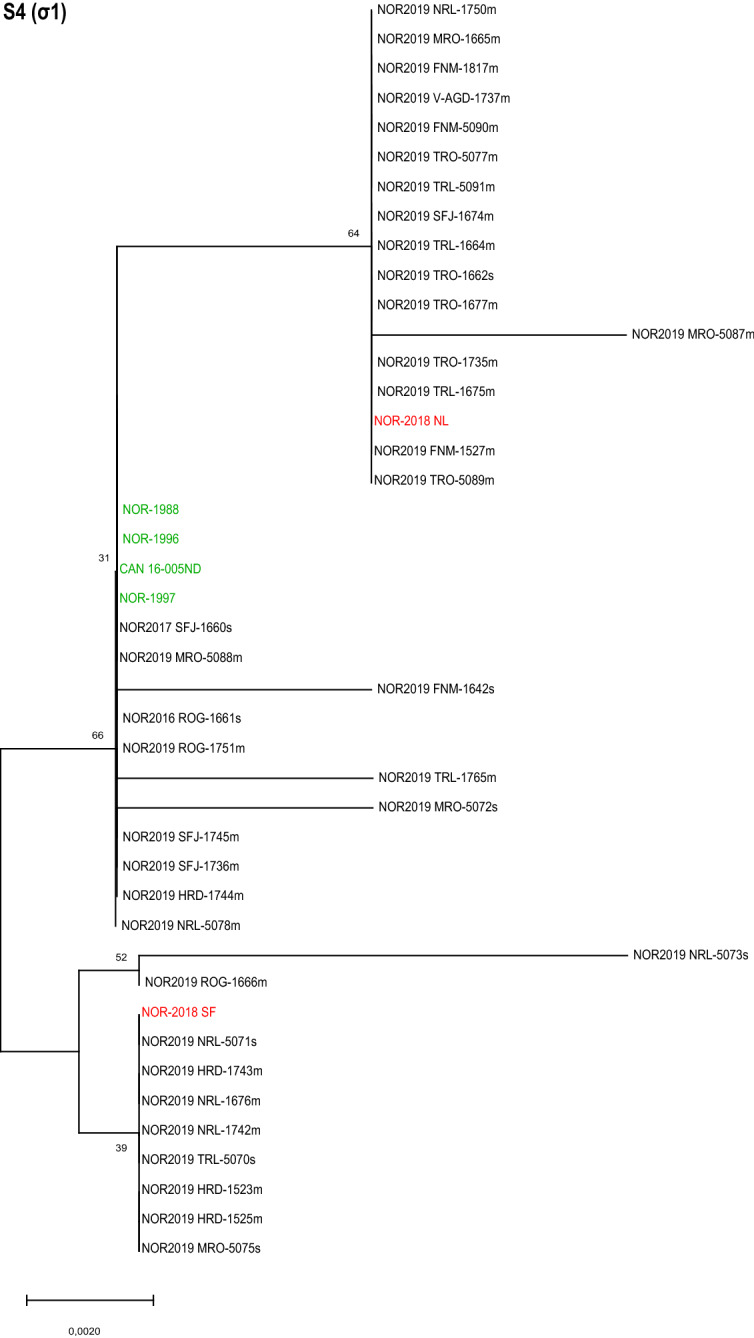
**Phylogenetic analysis of S4 (σ1).** Phylogenetic tree constructed from partial sequences of σ1 (aa 69–315) using neighbor joining (NJ). The analysis included 37 field isolates (black) and six reference isolates of known virulence (high virulent in red, low virulent in green). Bootstrap values were calculated from 1000 replicates and values above 70 were considered significant. The analysis did not reveal clustering with high enough bootstrap value to support distinct groups.

The phylogenetic analysis of the partial amino acid sequence σ1 (segment S4) did not reveal clustering with high enough bootstrap to support distinct groups (Figure 5). In terms of single amino acid substitutions, nine of the isolates had a V107A substitution, which is present in the high-virulent reference isolate NOR-2018 SF [4]. Sixteen of the isolates had a D252N substitution, which is found in the high-virulent reference isolate NOR-2018 NL [4]. These substitutions were not found simultaneously in any isolate, suggesting that they are mutually exclusive. These three groups, V107A, D252N and no substitution, were seen as three groups in the phylogenetic tree, but with bootstrap values below 70. This is likely due to other single amino acid substitutions present in several isolates. One field isolate differed from the other isolates in two amino acid positions, while four other field isolates had single amino acid substitutions not shared by any other isolates. No discernable patterns were found when analyzing the nucleotide sequence of segment S4 (Additional file 7).

### Eight different genogroups identified

Eight genogroups of PRV-1 were identified based on combinations of the five segments analyzed (Table 3). Two of the genogroups were defined as high-virulent based on identical segment combinations as the high-virulent reference isolates: High-1 (NOR-2018 NL) and High-2 (NOR-2018 NL). Two other genogroups were defined as low-virulent based on identical segment combinations as the low-virulent reference isolates: Low-1 (NOR-1997) and Low-2 (NOR-1996, NOR-1988 and CAN-16-005ND). Fourteen field isolates belonged to genogroup High-1, and nine isolates belonged to genogroup High-2. Seven field isolates belonged to genogroup Low-1 and only one field isolate belonged to the low-virulent genogroup Low-2.

**Table 3:** **Categorization of isolates into genogroups**

Genogroup (Reference isolate(s))	S1 (σ3) M2 (μ1)	L1 (λ3)	L2 (λ2)	S4 (σ1)	Isolates
High-1 (NOR-2018 NL)	Group B	Group A	Group A	D252N	NOR2019 TRO-1662s NOR2019 TRL-1675m NOR2019 TRL-1664m NOR2019 TRO-1677m NOR2019 MRO-1665m NOR2019 TRO-5077m NOR2019 MRO-5087m NOR2019-TRO-5089m NOR2019 FNM-5090m NOR2019 TRL-5091m NOR2019 FNM-1527m NOR2019 NRL-1750m NOR2019 TRO-1735m NOR2019 FNM-1817m
High-2 (NOR-2018 SF)	Group B	Group A	Group A	V107A	NOR2019 NRL-1676m NOR2019 NRL-1742m NOR2019 MRO-5075s NOR2019 TRL-5070s NOR2019 NRL-5071s NOR2019 NRL-5073s NOR2019 HRD-1743m NOR2019 HRD-1523m NOR2019 HRD-1525m
Low-1 (NOR-1997)	Group B	Group B	Group B	–	NOR2019 ROG-1666m NOR2016 ROG-1661s NOR2019 NRL-5078m NOR2019 FNM-1642s NOR2019 HRD-1744m NOR2019 ROG-1751m NOR2019 SFJ-1745m
Low-2 (NOR-1996, NOR-1988 CAN 16-005ND)	Group A	Group A	Group A	–	NOR2019 SFJ-1736m
Unknown-1 (None)	Group B	Group B	Group A	–	NOR2017 SFJ-1660s NOR2019 MRO-5072s NOR2019 MRO-5088m
Unknown-2 (None)	Group B	Group A	Group A	–	NOR2019 TRL-1765m
Unknown-3 (None)	Group B	Group B	Group A	D252N	NOR2019 SFJ-1674m
Unknown-4 (None)	Group A	Group A	Group A	D252N	NOR2019 V-AGD-1737m

The isolates were categorized into genogroups. The groping was based on the phylogenetic grouping (Group A or Group B) of S1 (σ3)/M2 (µ1), L1 (λ3), L2 (λ2) in addition to the presence of S4 (σ1) amino acid substitution V107A or D252N. Eight genogroups were identified including High-1, High-2 and Low-1, Low-2 with reference isolates of known virulence. In addition, four genogroups of unknown virulence were identified (Unknown 1–4). Isolates categorized to each genogroup listed on the right.

Four genogroups of unknown virulence with no reference isolates were found, annotated Unknown 1–4 (Table 3). Three field isolates grouped with Unknown-1. These isolates were similar to Low-1 except for L2 (λ2). One field isolate belonged to Unknown-3, which differed from Unknown-1 only in the S4 (σ1) D252N substitution. One field isolate had a segment composition highly similar to High-1 and High-2, but without substitutions in amino acid position 107 or 252 for S4 (σ1). This field isolate was annotated as Unknown-2. Finally, one field isolate shared a close similarity to Low-2. However, this field isolate had a S4 (σ1) D252N substitution and was therefore annotated Unknown-4.

### Geographic distribution of genogroups

**Figure 6:**
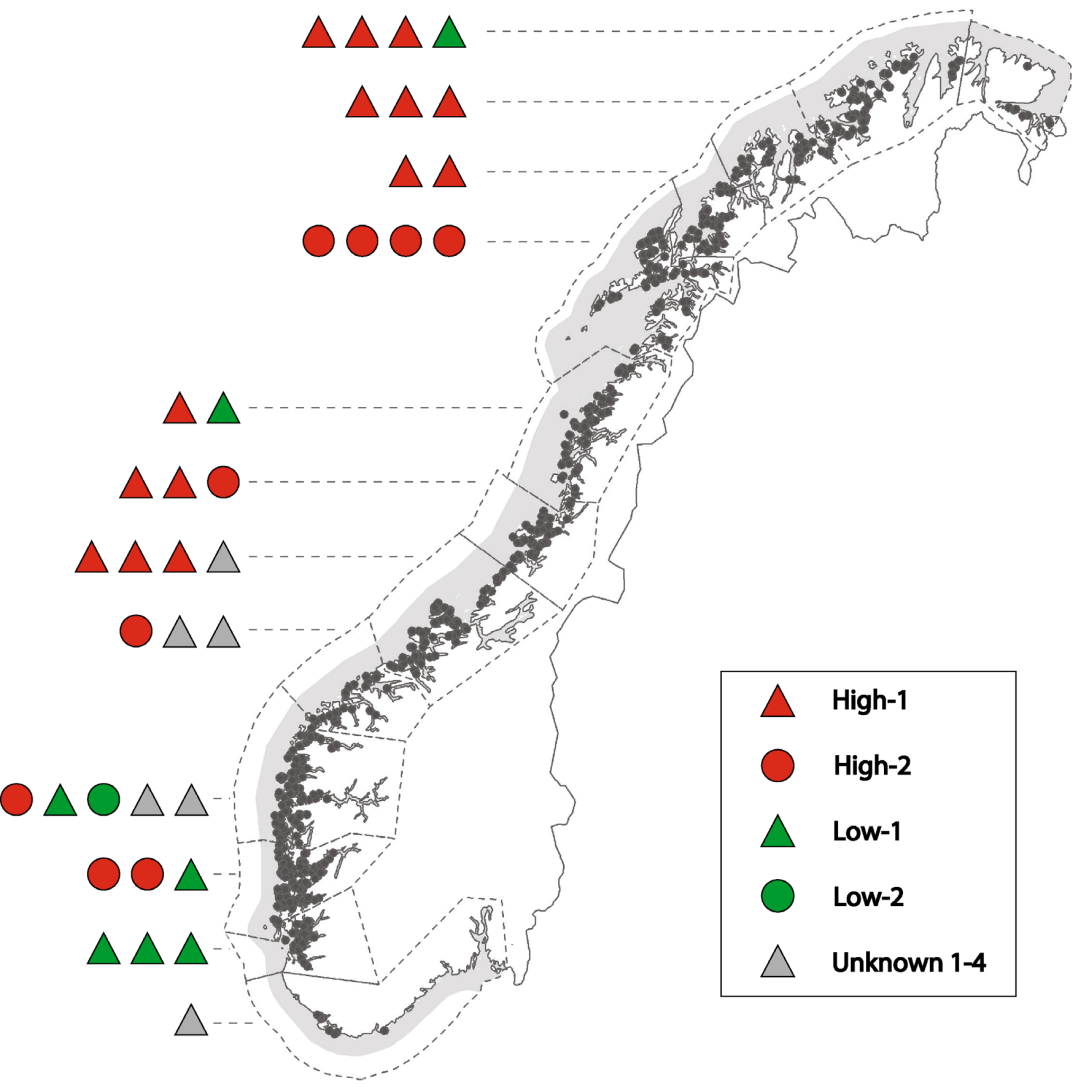
**Geographic distribution of genogroups.** Map created based on the geographic distribution of the genogroups of the 37 field isolates included in the study. Each field isolate was mapped according to genogroup and to the production zone from which it was collected. High- and low-virulent isolates were found in all regions, with a tendency for the high-virulent isolates to be more prevalent in the mid- and northern regions of Norway. Grey dashed lines indicate production zones.

The field isolates were mapped to production zones based on their genogroups to determine whether a geographic pattern of distribution could be determined (Figure 6). Overall, high- and low-virulent isolates were found in all regions. However, the mapping indicated a tendency for the high-virulent genogroups, High-1 and High-2, to be more prevalent in the mid- and northern regions of Norway. The southern region showed more diverse genetic grouping of PRV-1 including high-virulent, low-virulent and genogroups of unknown virulence.

## Discussion

The aim of the present study was to group PRV-1 variants present in Norwegian aquaculture based on their virulence properties. Due to their putative link to the virulence of PRV-1, the genomic segments S1, S4, M2, L1 and L2 and their encoded proteins were chosen as the basis for the categorization [4]. The sequences from 37 PRV-1 isolates collected from farmed Atlantic salmon in Norway were obtained, phylogenetic analysis of each segment was performed, and based on combinations of these five genomic segments eight genogroups were identified. Two groups were defined as high-virulent and two as low-virulent, whereas the remaining four groups were of unknown virulence. Previous phylogenetic studies have focused on nucleotide sequences, especially segment S1 [14, 16–18], but also partly on whole genome analysis [19, 28]. This has aided in description of the evolutionary relationships and geographical distribution of PRV-1. The present study focused on the encoded amino acid sequence, as virulence is most often linked to properties of viral proteins. We found that both high- and low-virulent isolates of PRV-1 circulate in farmed Atlantic salmon in Norway. The geographic distribution indicated a higher frequency of the high virulent isolates in the mid- and northern regions.

Previous phylogenetic analyses of segment S1 cluster PRV-1 into two main monophyletic groups, which has been referred to as Ia and Ib [16, 17]. However, the established classification of PRV-1 into Ia and Ib has important limitations with respect to virulence. The classification is based only on analysis of segment S1, but the S1 segment is not the sole determinant of virulence. Therefore, the Ia and Ib classification does not necessarily match with the virulence of the isolate. For example, it has been shown that low virulent variant NOR1997 has identical S1 sequence to that of high virulent variants (NOR-2018NL, NOR2018SF) [4]. Thus, other segments besides S1 are involved in determining the phenotype.

In the present study, the phylogenetic analysis of proteins σ3 in segment S1 confirmed the presence of two distinct clades consistent with the established classification. To emphasize that the analysis was on performed on the amino acid sequence in one specific segment the groups were annotated S1 (σ3) Group A and Group B. It should be noted that the amino acid focus may mask subtle sub-groupings compared to a nucleotide sequence analysis, but the approach highlights protein-differences which is likely to be the change linked to virulence.

A co-evolutionary relationship for S1 and M2 has been demonstrated earlier [19], and the present study confirmed the co-segregation of these two segments. Furthermore, S1 and M2 has been suggested as important for virulence [19]. However, the historical Norwegian isolate NOR-1997 has been shown to be of low-virulence in experimental challenge [4], even though it has identical S1 (σ3) and M2 (µ1) amino acid sequence to high-virulent reference isolates. This demonstrates that although S1 and M2 might be important, other segments and viral proteins are crucial for the viral virulence and that a classification system only based on S1 is not able to differentiate high and low virulent variants.

The unique L1- and L2 segments of the low-virulent NOR-1997 isolate suggest a potential role for these two segments in the virulence of the virus [4]. The L1 (λ3) and L2 (λ2) each divided the collection of field isolates into two groups, however no co-segregation of these two segments was observed. Most field isolates grouped with either Group A or Group B for both proteins, i.e., L1 (λ3) Group A/ L2 (λ2) Group A or L1 (λ3) Group B/ L2 (λ2) Group B. Only four isolates grouped with L1 (λ3) Group B and L2 (λ2) Group A, and no isolates were identified with the combination L1 (λ3) Group A and L2 (λ2) Group B. In contrast to S1 and M2, no studies have focused specifically on L1 and L2, and their contribution to virulence needs to be further investigated.

Phylogenetic analysis of σ1 encoded by segment S4 did not reveal groups with a high bootstrap value. Previous studies have indicated that confirmed low-virulent isolates have a valine residue in position 107 and an aspartic acid residue in position 252, while in contrast, confirmed high-virulent isolates vary in these positions [4]. Variations in these positions were mutually exclusive and grouped the field isolates into three distinct groups. The contribution of these substitutions to virulence requires further investigation. These amino acid variations can be a result of either direct mutations or recent reassortment events. We have therefore included them in the proposed genetic grouping system for PRV-1.

We propose a new extended system for categorization of PRV-1 isolates based on the amino acid sequences encoded by the five specific genomic segments earlier found to be related to virulence [4]. The categorization is based on the two distinct groups found for the four proteins S1 (σ3), M2 (µ1), L1 (λ3) and L2 (λ2), and single amino acid substitutions in positions 107 and 252 for σ1 in segment S4. The phylogenetic analysis used in the present study was based on partial sequences, enabling analysis of a higher number of isolates. The analyzed regions were selected because they differentiated between the reference strains of known virulence, which was used as a standard to characterize the field isolates [4]. However, single substitution or potential recombination events outside the studied region may be overlooked in this approach.

Reassortment of genetic segments is important for genetic change of mammalian reoviruses [22–24] and for the evolution of reoviruses in general, including PRV-1. Over the last decades, Norwegian aquaculture farming has become increasingly intensified. High fish density in the sea pens and the moving of fish and equipment over large geographic distances creates a melting pot for infectious agents and gives a selection pressure for genetic variants of viruses with increased fitness to the farming situation. This may in turn explain why the genogroups that dominate in Norwegian aquaculture farming today differ from those found decades ago, i.e., isolates NOR-1996 and NOR-1988. A basis for this development is the large genetic pool of PRV-1 found in Norway, exemplified by NOR-1997, which contributed with sequence diversity of segments L1 and L2, as well as S1 and M2. In contrast, the PRV-1 isolate on the North American Pacific coast appear homogenous [28], and this might limit the effect of reassortment events. In Norway, the natural host for PRV-1 is the wide-spread wild Atlantic salmon population, which over time probably has contributed to a large natural genetic pool for PRV-1 [14].

We observed that the reference isolates originally identified prior to the first clinical outbreak of HSMI, either belonged to Group A for all segments (NOR-1996 and NOR-1988) or Group B for all segments (NOR-1997). The contemporary high-virulent reference isolates (NOR-2018 SF and NOR-2018 NL) both contain a combination of the four segments from both Group A and Groups B. In line with this, the potential evolution from low- to high-virulent genogroups can tentatively be explained by reassortment of segments S1 and M2 of genogroup Low-1 (reference isolate NOR-1997), and L1 and L2 of genogroup Low-2 (reference isolates NOR-1996 and NOR-1988) to High-1 (reference isolate NOR-2018 NL) and High-2 (reference isolate NOR-2018 SF).

High-virulent genogroups dominate in Norwegian aquaculture farming today, however, low-virulent genogroups also circulate. The geographic mapping of the genogroups indicates that high-virulent isolates are more frequent in certain geographic regions in Norway. The first described clinical outbreak of HSMI in 1999 was observed in the mid region of Norway [29]. Although HSMI outbreaks are reported all along the coast of Norway, the mid and northern regions still appear to be among the most heavily affected areas. Fish health personnel from these regions consider HSMI to be a more serious disease problem than fish health personnel in the southern regions [8]. Although the disease is no longer notifiable, reports have indicated that outbreaks of HSMI are more common in these regions [8]. Our geographic mapping of the genogroups showed a tendency for a higher occurrence of the known high-virulent isolates in the middle and northern regions, which is coherent with the referred field observations.

The aim of the present study was to identify the different PRV-1 variants currently circulating in Norwegian Atlantic salmon aquaculture and to map their geographical distribution. To achieve this goal, sequence data from recently sampled PRV-1 isolates obtained from farmed Atlantic salmon with a known geographic location was essential. An additional requirement was that the samples had to include sequence information from all five PRV-1 genomic segments (S1, S4, M2, L1, L2) previously shown to be associated with virulence [4]. At the time of this study, a limited number of sequences were available in GenBank that meet these criteria. Including samples dating back longer in time, with non-specified geographic origin or lacking sequence from one or more segments would not serve the purpose of this study. This prompted us to collect a new set of sequences from farmed Atlantic salmon (35 samples from 2019, one sample from 2017, one sample from 2016) geographically covering the Norwegian coast. The study used six PRV-1 isolates with known virulence as framework for grouping the viruses into genogroups (referred to as reference isolates). These are the only PRV-1 isolates published in GenBank with a defined virulence based on data from a dose standardized challenge trial [4]. Although the aim of the present study restricted us from utilizing more sequences from GenBank, a similar approach would be applicable to identify PRV-1 variants in other geographic regions, from different time periods or to compare virus in wild and farmed Atlantic salmon.

The present study confirms circulation of both high- and low-virulent isolates of PRV-1 in farmed Atlantic salmon in Norway. Detection and differentiation between high- and low-virulent genogroups of PRV-1 could aid targeted disease control. A targeted effort against high-virulent variants could reduce the impact of PRV-1 related disease. The study also shows the existence of isolates of unknown virulence which should be characterized.

## Supplementary Information

**Additional file 1: Overview of amino acid differences between the reference isolates and the field isolates.**

**Additional file 2: Phylogenetic tree constructed from S1 (p13) using neighbor joining (NJ).** The analysis included 37 field isolates (black) and six reference isolates of known virulence (high virulent in red, low virulent in green). Bootstrap values were calculated from 1000 replicates.

**Additional file 3: Phylogenetic tree constructed from partial sequences of S1 (bp 77–1021) using Maximum Likelihood (ML).** The analysis included 37 field isolates (black) and six reference isolates of known virulence (high virulent in red, low virulent in green). Bootstrap values were calculated from 1000 replicates.

**Additional file 4: Phylogenetic tree constructed from partial sequences of M2 (bp 92–2108) using Maximum Likelihood (ML).** The analysis included 37 field isolates (black) and six reference isolates of known virulence (high virulent in red, low virulent in green). Bootstrap values were calculated from 1000 replicates.

**Additional file 5: Phylogenetic tree constructed from partial sequences of L1 (bp 2453–3877) using Maximum Likelihood (ML).** The analysis included 37 field isolates (black) and six reference isolates of known virulence (high virulent in red, low virulent in green). Bootstrap values were calculated from 1000 replicates.

**Additional file 6: Phylogenetic tree constructed from partial sequences of L2 bp (2332–3902) using Maximum Likelihood (ML).** The analysis included 37 field isolates (black) and six reference isolates of known virulence (high virulent in red, low virulent in green). Bootstrap values were calculated from 1000 replicates.

**Additional file 7: Phylogenetic tree constructed from partial sequences of S4 (bp 61–1006) using Maximum Likelihood (ML).** The analysis included 37 field isolates (black) and six reference isolates of known virulence (high virulent in red, low virulent in green). Bootstrap values were calculated from 1000 replicates.

## Abbreviations

Aa: amino acid
Bp: base pair
dsRNA: double-stranded RNA
HSMI: heart and skeletal muscle inflammation
PRV: Piscine orthoreovirus
WGS: whole genome sequencing
