# Supplementary material for: Genetic grouping and geographic distribution of Piscine orthoreovirus-1 (PRV-1) in farmed Atlantic salmon in Norway
Source: Vet Res. 2021 Oct 14;52:131. doi: 10.1186/s13567-021-01000-1 (PMC8515743; doi:10.1186/s13567-021-01000-1)
Supplement: Supplementary file 1 — Additional file 1: Overview of amino acid differences between the reference isolates and the field isolates. [file 13567_2021_1000_MOESM1_ESM.pdf]

	NOR-2018SF	NOR-2018NL	NOR-1997	NOR-1996	NOR-1988	CAN 16-005ND	Other
**L1 (λ3)** (pos 827-)	D_{937}	D_{937}	A_{937}	D_{937}	D_{937}	D_{937}	
	T_{939}	T_{939}	T_{939}	T_{939}	T_{939}	T_{939}	5089: A_{939}
	V_{962}	V_{962}	I_{962}	V_{962}	V_{962}	V_{962}	
	N_{996}	N_{996}	S_{996}	N_{996}	N_{996}	N_{996}	
	V_{1147}	V_{1147}	V_{1147}	V_{1147}	V_{1147}	V_{1147}	1743: G_{1147}
	V_{1265}	V_{1265}	I_{1265}	V_{1265}	V_{1265}	V_{1265}	
**L2 (λ2)** (pos 809-)	T_{932}	T_{932}	A_{932}	T_{932}	T_{932}	T_{932}	
	T_{1043}	T_{1043}	S_{1043}	N_{1043}	T_{1043}	T_{1043}	5087, 1738, 1743, 1675, 1677, 1665: A_{1043}<br>1735, 1737, 1817: N_{1043}
	V_{1055}	V_{1055}	I_{1055}	V_{1055}	V_{1055}	V_{1055}	
	D_{1071}	D_{1071}	E_{1071}	D_{1071}	D_{1071}	D_{1071}	
	I_{1075}	I_{1075}	I_{1075}	I_{1075}	I_{1075}	V_{1075}	All field isolates: I_{1075}
	A_{1128}	A_{1128}	A_{1128}	A_{1128}	A_{1128}	V_{1128}	All field isolates: A_{1128}
	P_{1141}	P_{1141}	L_{1141}	P_{1141}	P_{1141}	P_{1141}	
	G_{1279}	G_{1279}	G_{1279}	G_{1279}	G_{1279}	S_{1279}	All field isolates: G_{1279}
**S1 (σ3)** (pos 70-)	D_{78}	D_{78}	D_{78}	E_{78}	E_{78}	E_{78}	
	T_{85}	T_{85}	T_{85}	A_{85}	A_{85}	A_{85}	
	T_{90}	T_{90}	T_{90}	T_{90}	T_{90}	T_{90}	1735: A_{90}
	N_{117}	N_{117}	N_{117}	T_{117}	T_{117}	T_{117}	
	V_{136}	V_{136}	V_{136}	I_{136}	I_{136}	I_{136}	
	T_{156}	T_{156}	T_{156}	A_{156}	A_{156}	A_{156}	
	A_{157}	A_{157}	A_{157}	S_{157}	S_{157}	S_{157}	
	E_{174}	E_{174}	E_{174}	K_{174}	K_{174}	K_{174}	
	S_{180}	S_{180}	S_{180}	S_{180}	S_{180}	L_{180}	All field isolates: S_{180}
	T_{200}	T_{200}	T_{200}	T_{200}	T_{200}	T_{200}	1674, 1675: H_{200}
	A_{206}	A_{206}	A_{206}	V_{206}	V_{206}	V_{206}	
	V_{218}	V_{218}	V_{218}	I_{218}	I_{218}	I_{218}	
	T_{226}	T_{226}	T_{226}	T_{226}	T_{226}	T_{226}	1750: A_{226}
	V_{230}	V_{230}	V_{230}	V_{230}	V_{230}	A_{230}	All field isolates: V_{230}
	A_{267}	A_{267}	A_{267}	A_{267}	A_{267}	A_{267}	5091: V_{267}
**M2 (µ1)** (pos 47-)	T_{184}	T_{184}	T_{184}	S_{184}	S_{184}	S_{184}	
	T_{227}	T_{227}	T_{227}	T_{227}	T_{227}	T_{227}	1742: A_{227}
	S_{262}	S_{262}	S_{262}	A_{262}	A_{262}	A_{262}	
	A_{320}	A_{320}	A_{320}	A_{320}	A_{320}	A_{320}	1737, 1674: T_{320}
	D_{370}	D_{370}	D_{370}	N_{370}	N_{370}	N_{370}	
	A_{371}	A_{371}	A_{371}	A_{371}	A_{371}	A_{371}	1743: V_{371}
	V_{389}	V_{389}	V_{389}	V_{389}	A_{389}	V_{389}	All field isolates: V_{389}
	S_{560}	S_{560}	S_{560}	S_{560}	S_{560}	S_{560}	5078: P_{560}
	K_{674}	K_{674}	K_{674}	K_{674}	K_{674}	K_{674}	5090: R_{674}
**S4 (σ1)** (pos 69-)	A_{99}	A_{99}	A_{99}	A_{99}	A_{99}	A_{99}	5073: D_{99}
	A_{107}	V_{107}	V_{107}	V_{107}	V_{107}	V_{107}	
	I_{114}	I_{114}	I_{114}	I_{114}	I_{114}	I_{114}	1642: T_{114}
	V_{121}	V_{121}	V_{121}	V_{121}	V_{121}	V_{121}	5072: M_{121}
	D_{134}	D_{134}	D_{134}	D_{134}	D_{134}	D_{134}	1675: G_{134}
	V_{146}	V_{146}	V_{146}	V_{146}	V_{146}	V_{146}	5087: A_{146}
	T_{164}	T_{164}	T_{164}	T_{164}	T_{164}	T_{164}	5073: S_{164}
	D_{252}	N_{252}	D_{252}	D_{252}	D_{252}	D_{252}	
