# Supplementary figures and images for: Genetic grouping and geographic distribution of Piscine orthoreovirus-1 (PRV-1) in farmed Atlantic salmon in Norway
Source: Vet Res. 2021 Oct 14;52:131. doi: 10.1186/s13567-021-01000-1 (PMC8515743; doi:10.1186/s13567-021-01000-1)

S1 (p13)

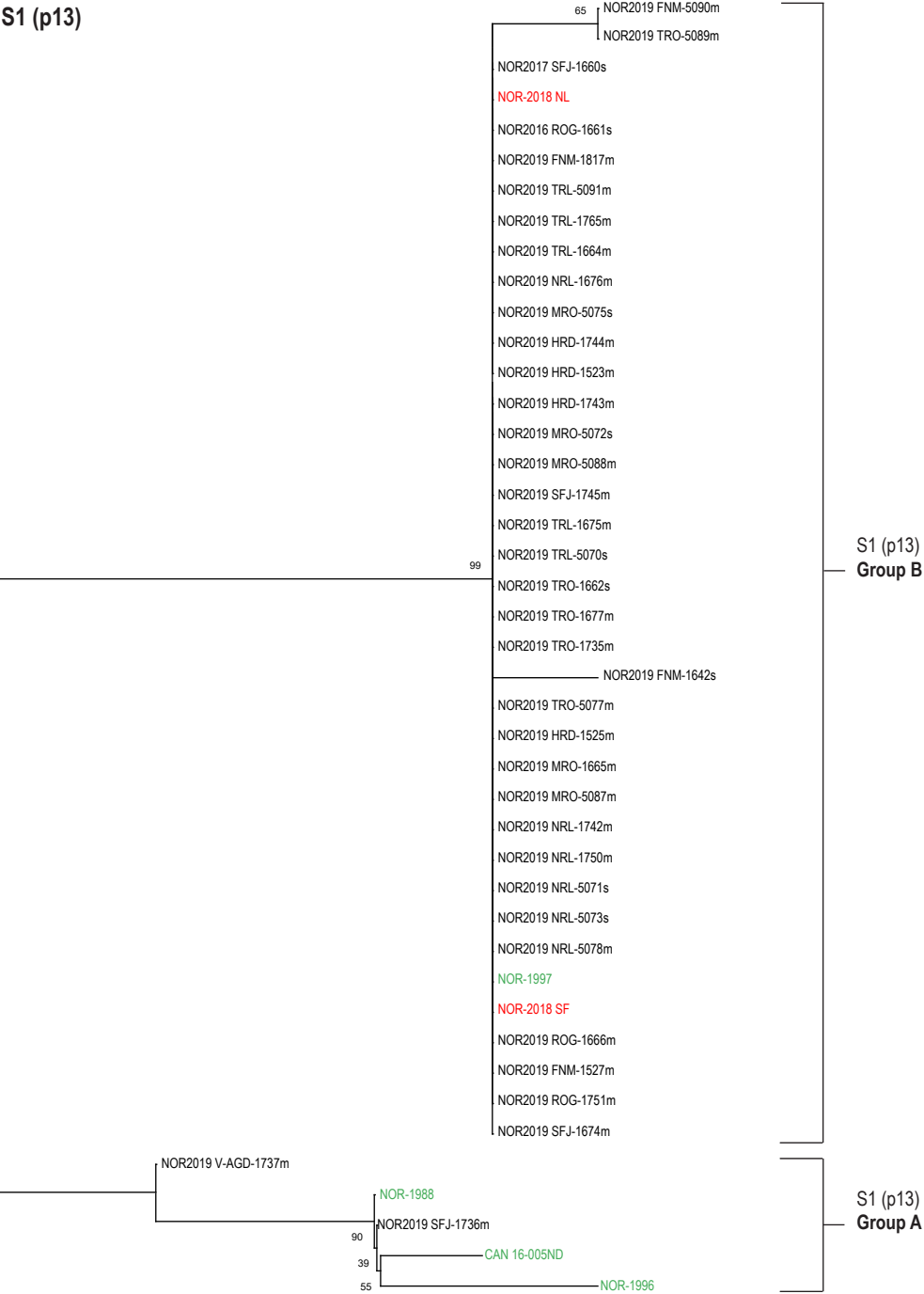

Supplement: Supplementary file 2 — Additional file 2: Phylogenetic tree constructed from S1 (p13) using neighbor joining (NJ). The analysis included 37 field isolates (black) and six reference isolates of known virulence (high virulent in red, low virulent in green). Bootstrap values were calculated from 1000 replicates. [file 13567_2021_1000_MOESM2_ESM.pdf]

Segment S1

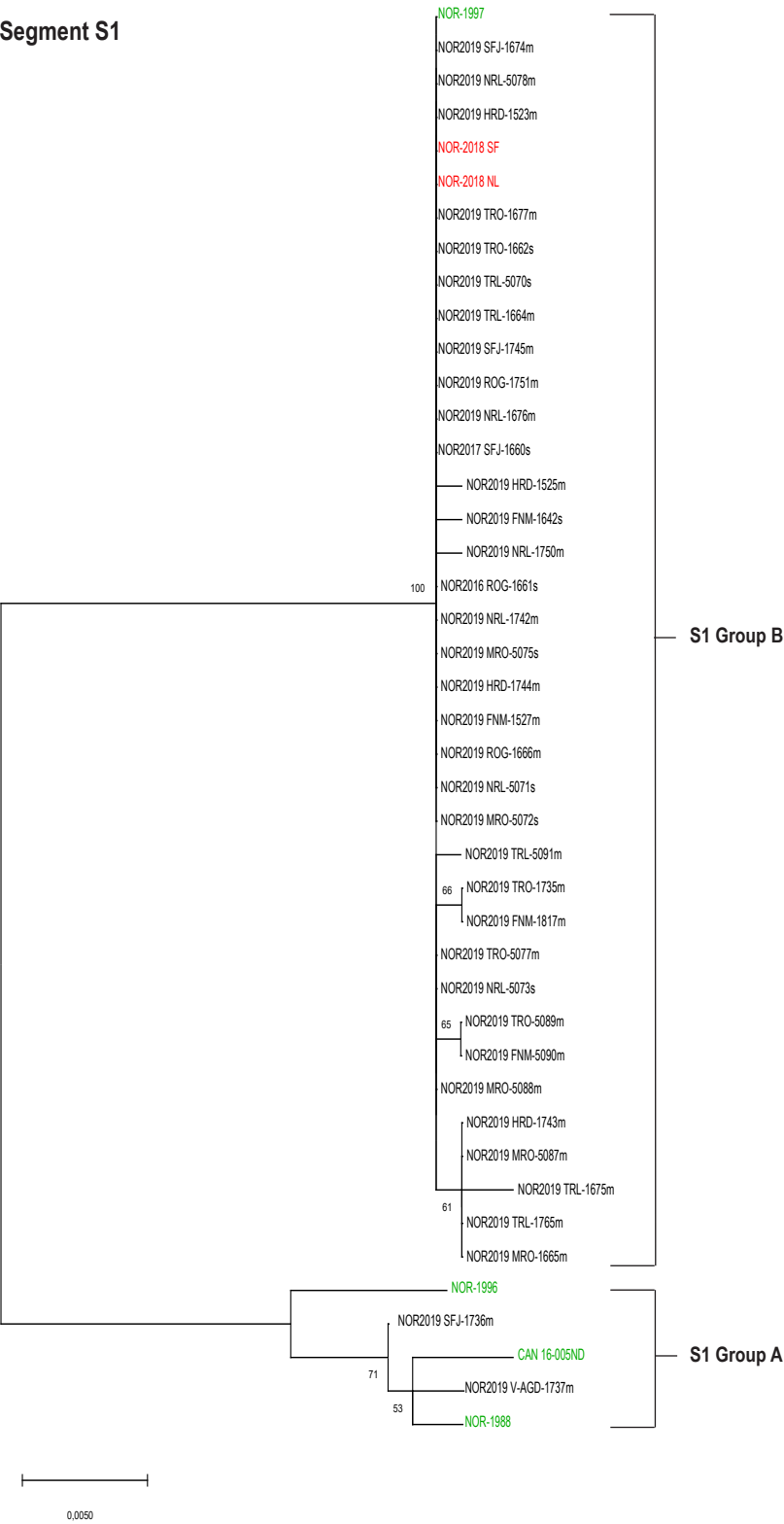

Supplement: Supplementary file 3 — Additional file 3: Phylogenetic tree constructed from partial sequences of S1 (bp 77–1021) using Maximum Likelihood (ML). The analysis included 37 field isolates (black) and six reference isolates of known virulence (high virulent in red, low virulent in green). Bootstrap values were calculated from 1000 replicates. [file 13567_2021_1000_MOESM3_ESM.pdf]

Segment M2

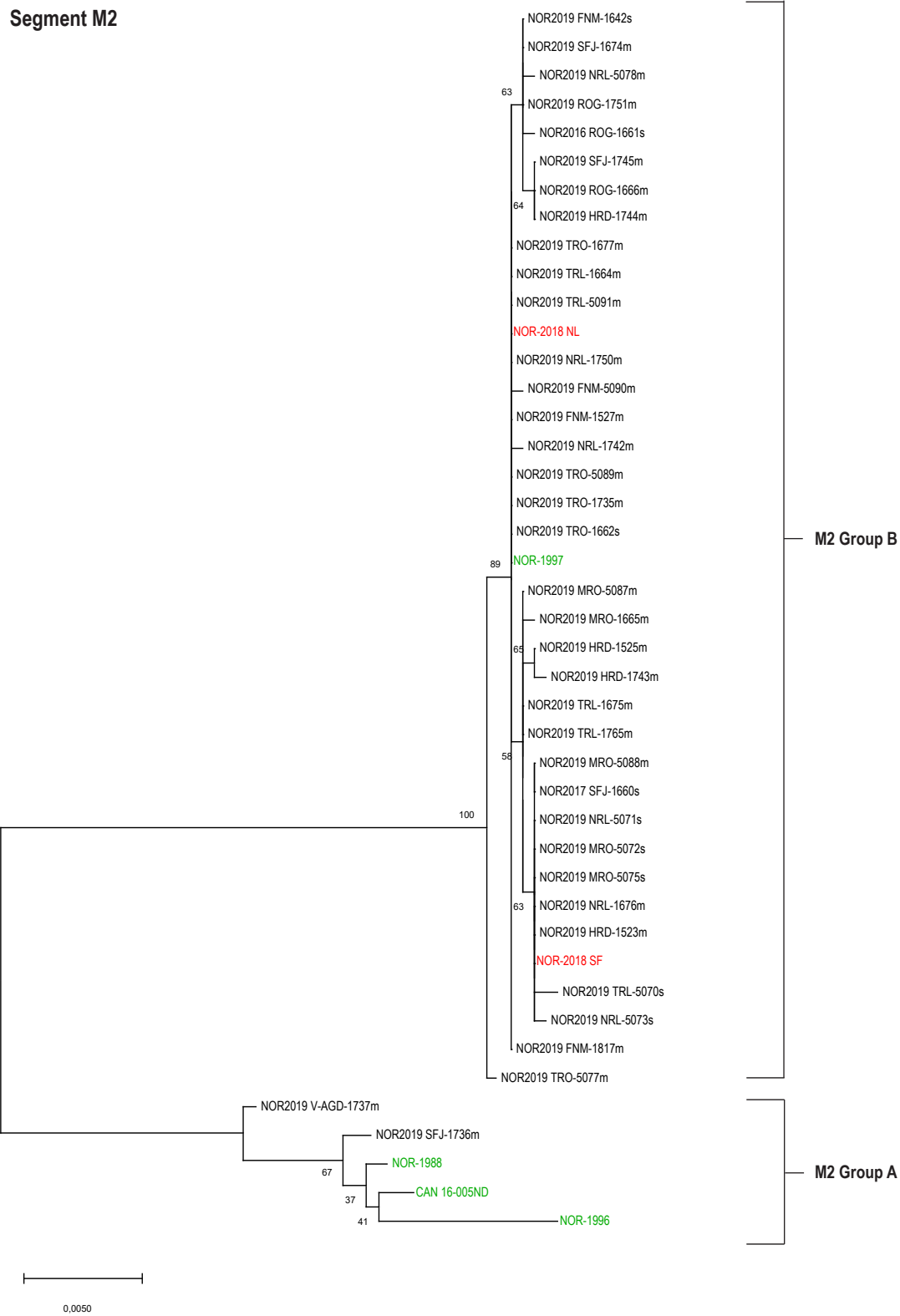

Supplement: Supplementary file 4 — Additional file 4: Phylogenetic tree constructed from partial sequences of M2 (bp 92–2108) using Maximum Likelihood (ML). The analysis included 37 field isolates (black) and six reference isolates of known virulence (high virulent in red, low virulent in green). Bootstrap values were calculated from 1000 replicates. [file 13567_2021_1000_MOESM4_ESM.pdf]

Segment L1

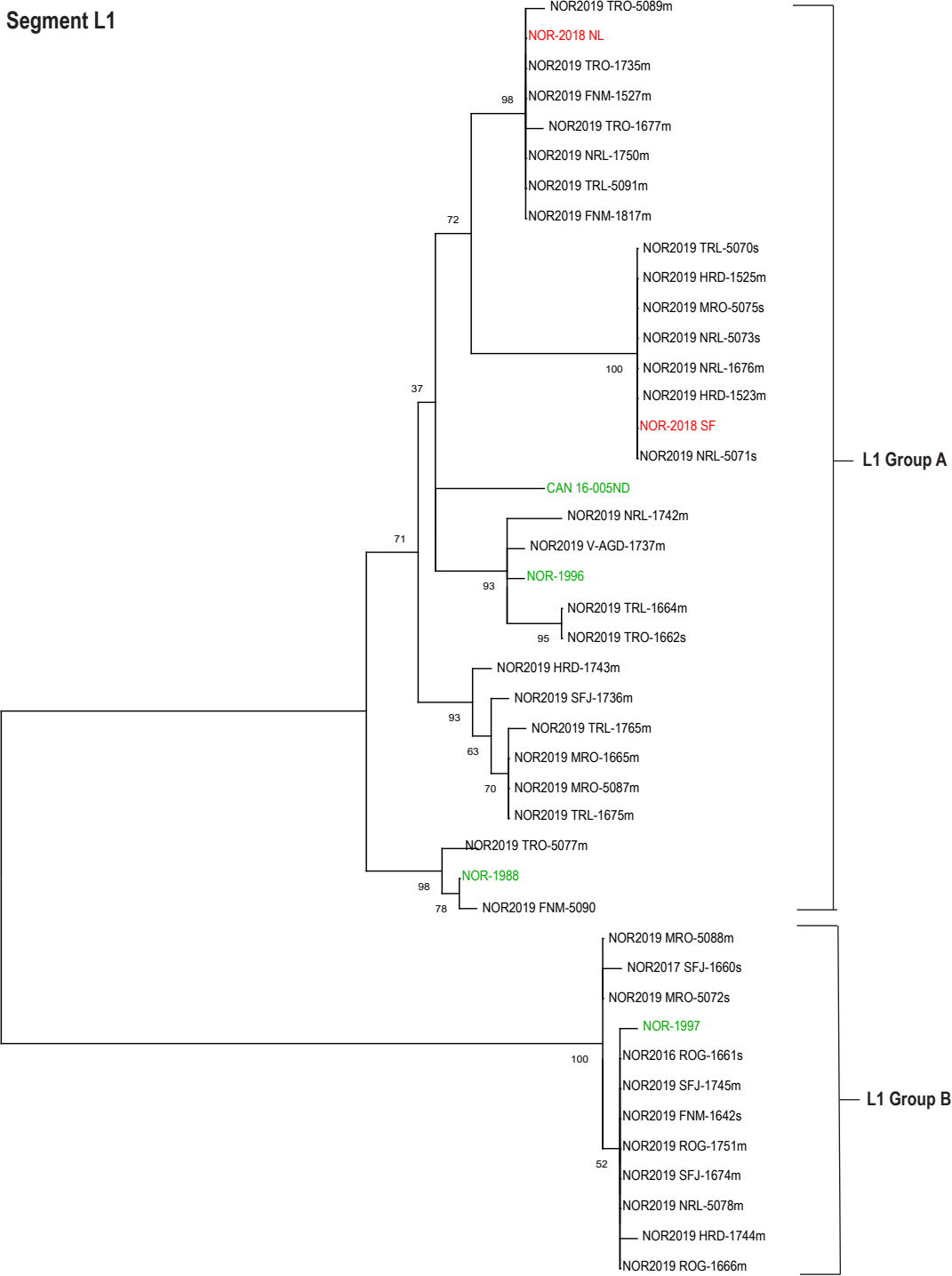

Supplement: Supplementary file 5 — Additional file 5: Phylogenetic tree constructed from partial sequences of L1 (bp 2453–3877) using Maximum Likelihood (ML). The analysis included 37 field isolates (black) and six reference isolates of known virulence (high virulent in red, low virulent in green). Bootstrap values were calculated from 1000 replicates. [file 13567_2021_1000_MOESM5_ESM.pdf]

Segment L2

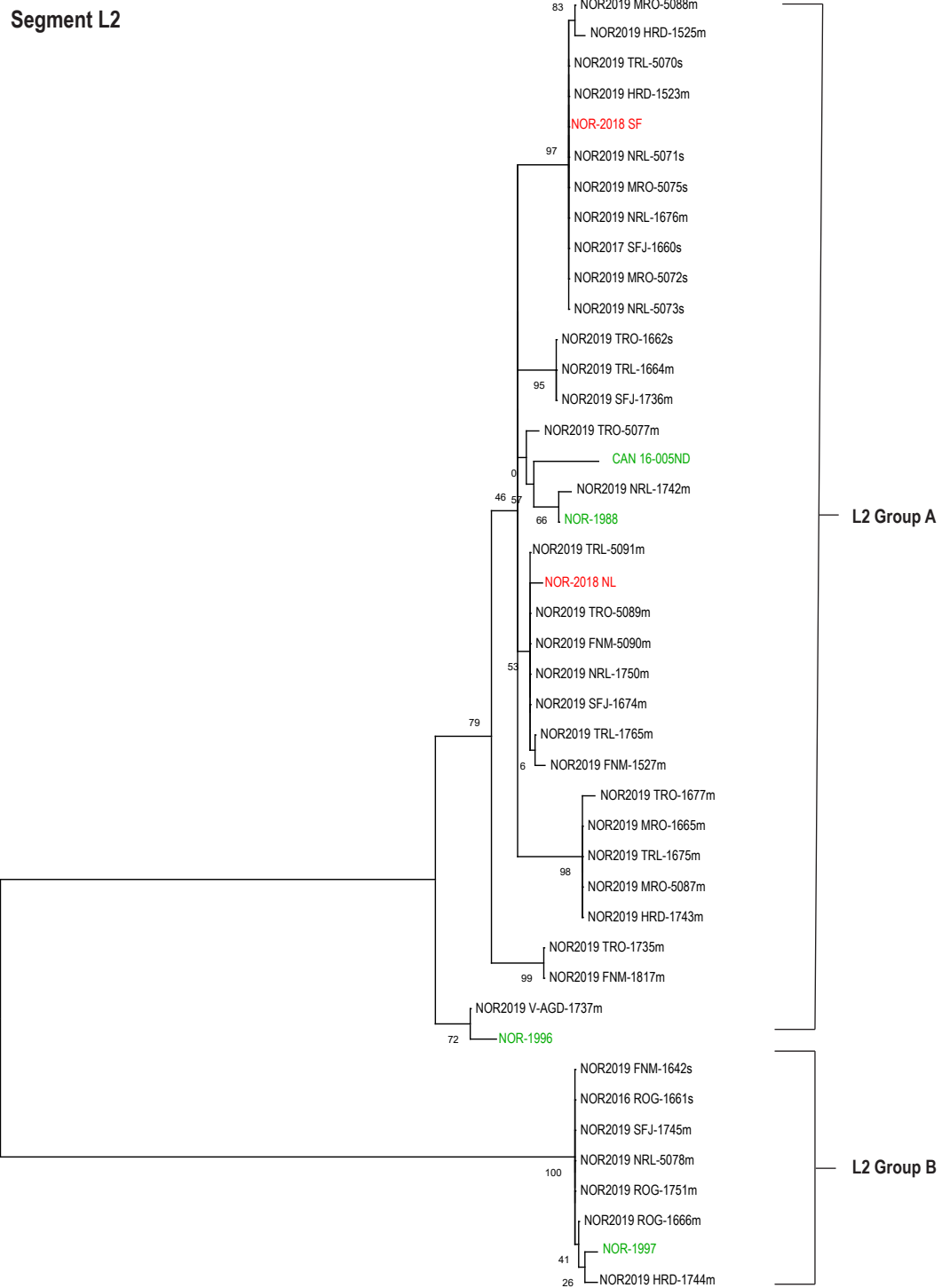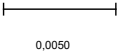

Supplement: Supplementary file 6 — Additional file 6: Phylogenetic tree constructed from partial sequences of L2 bp (2332–3902) using Maximum Likelihood (ML). The analysis included 37 field isolates (black) and six reference isolates of known virulence (high virulent in red, low virulent in green). Bootstrap values were calculated from 1000 replicates. [file 13567_2021_1000_MOESM6_ESM.pdf]

Segment S4

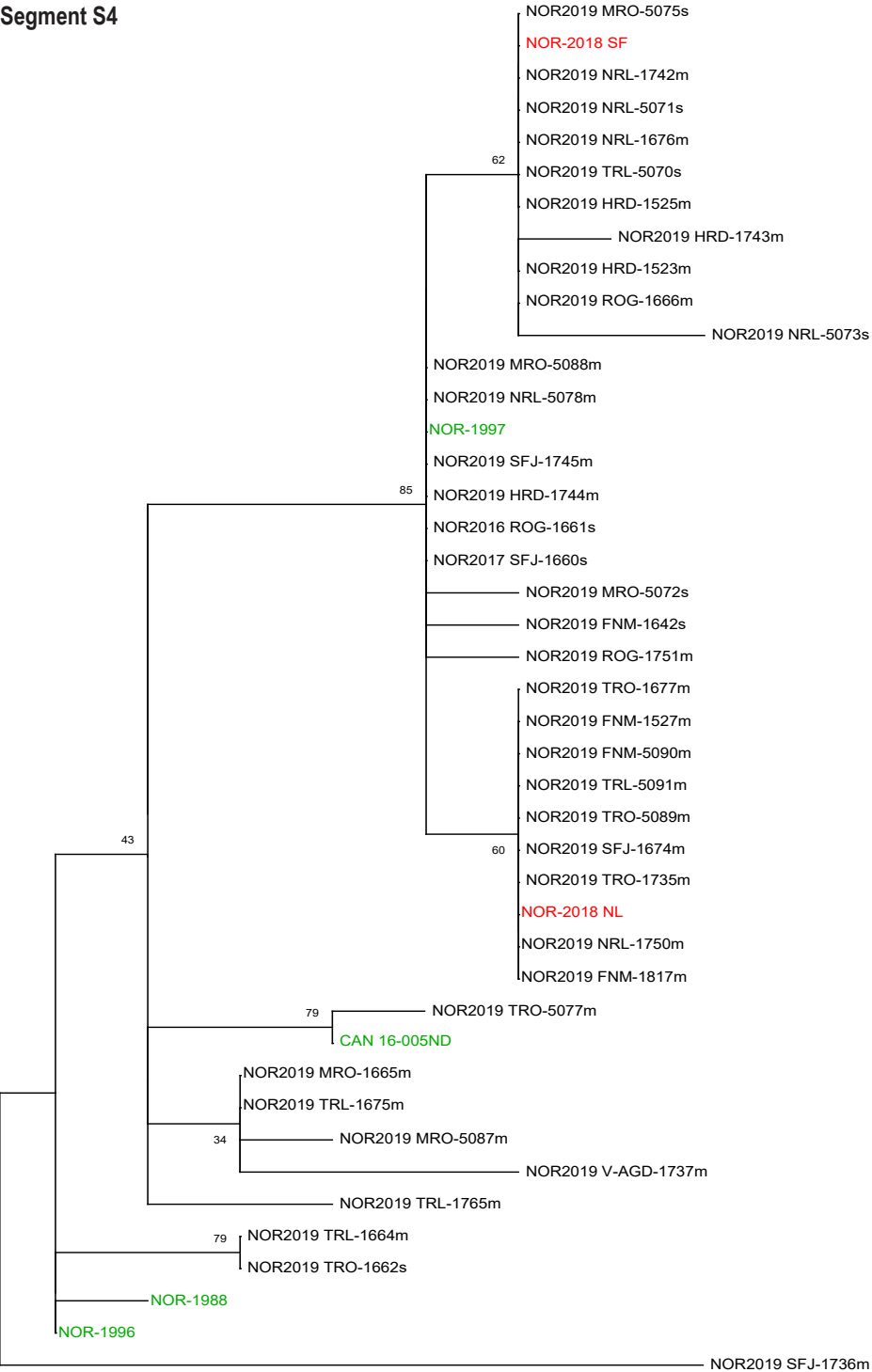

Supplement: Supplementary file 7 — Additional file 7: Phylogenetic tree constructed from partial sequences of S4 (bp 61–1006) using Maximum Likelihood (ML). The analysis included 37 field isolates (black) and six reference isolates of known virulence (high virulent in red, low virulent in green). Bootstrap values were calculated from 1000 replicates. [file 13567_2021_1000_MOESM7_ESM.pdf]
